# Characterization of T-Cell Responses to Conserved Regions of the HIV-1 Proteome in BALB/c Mice

**DOI:** 10.1128/CVI.00587-14

**Published:** 2014-11

**Authors:** Beatrice Ondondo, Sultan Abdul-Jawad, Anne Bridgeman, Tomáš Hanke

**Affiliations:** a The Jenner Institute, Nuffield Department of Medicine, University of Oxford, Oxford, United Kingdom; b MRC Human Immunology Unit, Weatherall Institute of Molecular Medicine, Nuffield Department of Medicine, University of Oxford, The John Radcliffe, Oxford, United Kingdom

## Abstract

A likely requirement for a protective vaccine against human immunodeficiency virus type 1 (HIV-1)/AIDS is, in addition to eliciting antibody responses, induction of effective T cells. To tackle HIV-1 diversity by T-cell vaccines, we designed an immunogen, HIVconsv, derived from the most functionally conserved regions of the HIV-1 proteome and demonstrated its high immunogenicity in humans and rhesus macaques when delivered by regimens combining plasmid DNA, nonreplicating simian (chimpanzee) adenovirus ChAdV-63, and nonreplicating modified vaccinia virus Ankara (MVA) as vectors. Here, we aimed to increase the decision power for iterative improvements of this vaccine strategy in the BALB/c mouse model. First, we found that prolonging the period after the ChAdV63.HIVconsv prime up to 6 weeks increased the frequencies of HIV-1-specific, gamma interferon (IFN-γ)-producing T cells induced by the MVA.HIVconsv boost. Induction of strong responses allowed us to map comprehensively the H-2^d^-restricted T-cell responses to these regions and identified 8 HIVconsv peptides, of which three did not contain a previously described epitope and were therefore considered novel. Induced effector T cells were oligofunctional and lysed sensitized targets *in vitro*. Our study therefore provides additional tools for studying and optimizing vaccine regimens in this commonly used small animal model, which will in turn guide vaccine improvements in more expensive nonhuman primate and human clinical trials.

## INTRODUCTION

The quest for a safe and effective vaccine against human immunodeficiency virus type 1 (HIV-1)/AIDS continues (1). Both prophylactic and particularly therapeutic vaccines will likely require induction of effective cytotoxic CD8^+^ T cells in addition to protective antibodies. There is strong evidence showing that HIV-1-specific CD8^+^ T cells contribute to the control of HIV-1 replication during acute and chronic stages of infection by killing virus-infected cells and by producing a number of soluble factors with antiviral activities (2). However, the initial CD8^+^ T-cell response, though strong, is typically directed toward a few immunodominant variable epitopes (3) often driving selection of virus escape mutations (4–6) and substantially contributing to the evolution of a large number of HIV-1 quasispecies detected in most infected individuals (7).

To tackle HIV-1 diversity and escape, we designed a novel immunogen, HIVconsv, assembled from the 14 most conserved regions of the HIV-1 proteome and encompassing consensus amino acid sequences derived from the four major alternating HIV-1 clades A, B, C, and D (8, 9). This immunogen was presented to the immune system using a variety of vaccine vectors such as plasmid DNA with and without electroporation, human and simian adenoviruses, poxvirus modified vaccinia virus Ankara (MVA), alphavirus Semliki Forest virus replicons, and modalities such as adjuvanted synthetic long peptides (9–15). These HIVconsv vaccines were used as a standalone delivery and more often in heterologous prime-boost regimens to enhance transgene product-specific responses while avoiding boost of responses against the delivery vectors. In human studies, conserved regions delivered by a combination of plasmid DNA pSG2.HIVconsv, simian (chimpanzee) adenovirus ChAdV63.HIVconsv, and MVA.HIVconsv elicited high frequencies of oligofunctional T-cell responses with broad specificities, which correlated with inhibition of 2 out of 8 tested HIV-1 isolates in an *in vitro* HIV-1 inhibition assay in the majority of vaccine recipients (16). While these initial preclinical and phase I clinical trial results are highly encouraging for the conserved region strategy, there is room for improvement, for example, in terms of the breadth of HIV-1 variant inhibition. Thus, vaccine modalities, conserved immunogen designs, regimens, routes of delivery, and adjuvantation will need to be modified and tested first in iterative preclinical studies to improve the vaccine performance.

To date, we have used predominantly a single immunodominant CD8^+^ T-cell epitope, H-2D^d^- and L^d^-restricted RGPGRAFVTI, designated P18-I10 (17, 18) or historically by us as the H epitope (19), which was added to the C terminus of candidate HIV-1-derived immunogens HIVA and HIVconsv to inform vaccine development in the BALB/c mouse model (9, 20). The study presented here describes powering of this model for further vaccine and regimen improvements by detailed mapping of vaccine-induced T-cell specificities supported by functional characterization of the HIVconsv vaccine-induced cellular responses, which provides comprehensive and sensitive tools for further vaccine advances.

## MATERIALS AND METHODS

### Mouse immunizations.

Six-week-old female BALB/c mice were immunized intramuscularly with doses of recombinant ChAdV63.HIVconsv or recombinant MVA.HIVconsv as indicated for each experiment. Animal care and procedures conformed to the United Kingdom Home Office Guidelines under the Animals (Scientific Procedures) Act 1986. The protocol was approved by the local Research Ethics Committee (Clinical Medicine, University of Oxford). Experiments were carried out under project license no. 30/2833 held by T.H. with a strict implementation of the Replacement, Reduction, and Refinement (3Rs) principles.

### Preparation of splenocytes.

Spleens were collected, and cells were isolated by pressing organs individually through a 70-μm nylon cell strainer (BD Falcon) using a 5-ml syringe rubber plunger. Following the removal of red blood cells with RBC Lysing Buffer Hybri-Max (Sigma), splenocytes were washed and suspended in R10 (RPMI 1640 supplemented with 10% fetal calf serum [FCS], penicillin-streptomycin, and β-mercaptoethanol).

### Peptides and peptide pools.

One hundred ninety-nine HIVconsv-derived peptides (15/11) were divided into 6 pools of 32 to 35 individual peptides and were used at a final concentration of 1.5 μg/ml in all assays as described previously (16). Groups of truncated peptides were treated identically. All peptides were synthesized by GenScript HK Limited (Hong Kong), purified to ≥90% purity, and confirmed by high-performance liquid chromatography (HPLC)–mass spectrometry.

### IFN-γ ELISPOT assay.

The enzyme-linked immunospot (ELISPOT) assay was performed using the mouse gamma interferon (IFN-γ) ELISPOT kit (Mabtech) according to the manufacturer's instructions. Spots were visualized using sequential applications of a biotin-conjugated secondary anti-IFN-γ monoclonal antibody (MAb) (R4-6A2, rat IgG1), alkaline phosphatase, and a chromogenic substrate (Bio-Rad) and counted using the AID ELISpot reader system (Autoimmun Diagnostika). While all 15-mer peptides were tested on cells from individual mice, optimal epitope mapping employed pooled samples.

### Intracellular cytokine staining (ICS).

One million splenocytes or pooled peripheral blood mononuclear cells (PBMCs) were stimulated with peptides or peptide pools at 37°C and 5% CO_2_ for 90 min, before addition of Golgi Stop (BD Bioscience). CD107a-fluorescein isothiocyanate (FITC) antibody was added at the start of stimulation. After a 5-h incubation, the cells were washed with fluorescence-activated cell sorting (FACS) buffer (phosphate-buffered saline [PBS], 1% FCS, 0.01% azide), blocked with anti-CD16/32 antibodies (eBioscience) at 4°C for 20 min, and then stained with anti-CD8 MAb (eBioscience). The cells were washed, permeabilized, and stained for intracellular cytokines: anti-tumor necrosis factor alpha (anti-TNF-α), anti-IFN-γ, and anti-interleukin-2 (anti-IL-2) MAbs (eBioscience). Following a wash and fixation, the cells were acquired using an LSR II flow cytometer (BD Biosciences) and analyzed with the FlowJo (Tree Star) and SPICE programs.

### *Ex vivo* killing assay.

Equal numbers of P815 target cells were differentially labeled with either 800 nM or 32 nM carboxyfluorescein succinimidyl ester (CFSE) according to the manufacturer's specifications. The P815 cells labeled with 800 nM CFSE were pulsed with peptides for 2 h and washed several times. Splenocytes from immunized mice were prepared as described above, mixed with the differentially CFSE-labeled target cells at an effector-to-target (ET) ratio of 10:1 or 5:1, and incubated overnight at 37°C. The cells were washed, stained with a LIVE/DEAD marker, and analyzed using flow cytometry. Cytotoxicity was calculated as follows: % specific lysis = 100 × (number of unpulsed control cells − number of peptide-pulsed cells)/number of unpulsed control cells.

### Statistical analysis.

Statistical analyses were performed using Prism 6 for Mac X version 6. Multiple comparisons utilized one-way analysis of variance (ANOVA), while group pairs were compared using the two-tailed unpaired *t* test with Welch's correction. A *P* value of <0.05 was considered significant.

## RESULTS

### A longer interval after ChAdV63.HIVconsv administration benefits MVA.HIVconsv boost.

**FIG 1:**
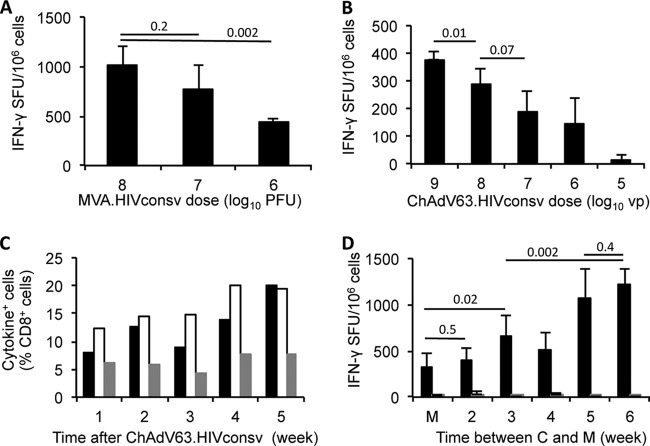
Optimization of ChAdV63.HIVconsv-MVA.HIVconsv vaccine delivery. (A and B) Groups of BALB/c mice were immunized with indicated doses of either MVA.HIVconsv (A) or ChAdV63.HIVconsv (B), and responses to peptide H were assessed in an IFN-γ ELISPOT assay after 1 or 3 weeks, respectively. The dosage groups were significantly different with *P* = 0.011, *n* = 5 (A), and *P* < 0.0001, *n* = 4 (B). (C) Mice received a single injection of 10^8^ vp of ChAdV63.HIVconsv, and the induction of IFN-γ (black bars)-, CD107a (white bars)-, and TNF-α (gray bars)-positive cells at various time points after vaccine administration was determined using pooled PBMCs in an ICS assay after stimulation with peptide H. (D) The same mice were boosted with 10^6^ PFU of MVA.HIVconsv 1 week after the bleed, and control naive mice received MVA.HIVconsv alone without prior priming (M). Peptide H (black bars)- and G1 (AMQMLKDTI; gray bars)-specific splenocytes were enumerated in an IFN-γ ELISPOT assay 1 week after administration of MVA.HIVconsv. Groups are significantly different with *P* < 0.0001, *n* = 4. Data for peptide H in panels A, B, and D were analyzed using one-way ANOVA, and column pairs were compared using a two-tailed unpaired *t* test with Welch's correction.

BALB/c mice were immunized with single decreasing doses of MVA.HIVconsv (Fig. 1A) or ChAdV63.HIVconsv (Fig. 1B), and the splenocytes were tested in immunogenicity assays after 1 or 3 weeks, respectively. Immunogenicity was measured by the frequency of HIVconsv-induced T cells recognizing the immunodominant H-2K^d^/H-2L^d^-restricted HIV-1 epitope H originating from the hypervariable loop 3 of Env (residues 311 to 320) (18, 19) in an IFN-γ ELISPOT assay. We found that the frequency of peptide H-specific T cells increased with increasing dose of MVA.HIVconsv, and at the highest dose of 10^8^ PFU, an average of 1,016 IFN-γ spot-forming units (SFU)/10^6^ splenocytes were detected (Fig. 1A). Likewise, following ChAdV63.HIVconsv immunization, the frequency of IFN-γ-producing cells increased steadily with increasing vaccine dose and reached an average of 378 SFU/10^6^ splenocytes at a dose of 10^9^ virus particles (vp) (Fig. 1B). For subsequent experiments, ChAdV63.HIVconsv and MVA.HIVconsv vaccines were used at 10^8^ vp and 10^6^ PFU, respectively; we did not choose the maximum doses to avoid saturating the system, which might obscure detection of possible enhancements.

The overall magnitude of responses at 1 week after ChAdV63.HIVconsv immunization was much lower than that after MVA.HIVconsv immunization. We therefore sought to define the optimal time for achieving a peak multifunctional response following ChAdV63.HIVconsv administration. Groups of mice were immunized with a single dose of 10^8^ vp of ChAdV63.HIVconsv, and peptide H-specific T-cell responses in pooled PBMCs were determined using an intracellular cytokine staining (ICS) assay for IFN-γ, CD107a, and TNF-α, at 1 to 5 weeks postimmunization. Indeed, vaccine-specific T-cell frequencies peaked at 20% of IFN-γ^+^ CD8^+^ cells of total CD8^+^ cells at week 5 after immunization (Fig. 1C), suggesting prolonged antigenic stimulation, possibly due to persistence of low levels of transcriptionally active ChAdV63.HIVconsv genomes, as previously described (21). One week after the bleed, the mice were boosted with 10^6^ PFU MVA.HIVconsv to assess the impact of the interval between the ChAdV63.HIVconsv prime and the MVA.HIVconsv boost on the magnitude of T-cell responses. A strong synergistic effect between the two vaccines, which peaked at 1,217 SFU/10^6^ splenocytes at a 6-week gap, was observed (Fig. 1D). These experiments indicated that a 5- to 6-week interval between the ChAdV63.HIVconsv prime and MVA.HIVconsv boost should be used for eliciting high frequencies of T-cell responses, which will in turn allow detailed mapping of subdominant epitopes. Subsequent experiments were thus performed using a 6-week interval.

### Specificity mapping of HIVconsv-elicited T cells.

**FIG 2:**
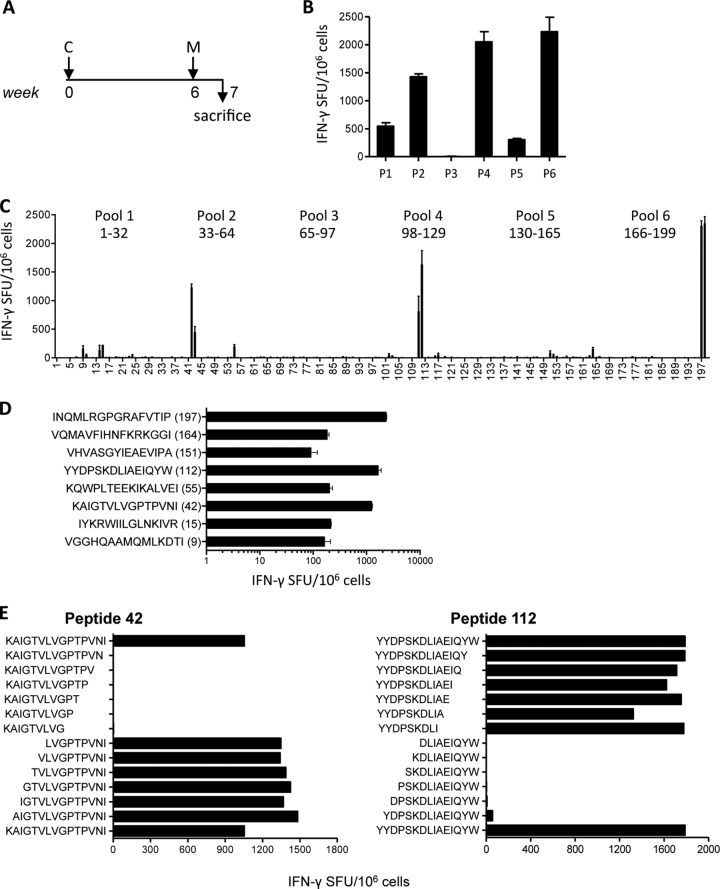
Breadth, magnitude, and fine specificity of T-cell responses induced by the HIVconsv vaccines. (A) Immunization schedule of BALB/c mice: C and M represent 10^8^ vp of ChAdV63.HIVconsv and 10^6^ PFU of MVA.HIVconsv, respectively. (B and C) Splenocytes were tested for IFN-γ production in an ELISPOT assay upon restimulation with peptide pools P1 to P6 (B) or individual 15/11 HIVconsv peptides derived from the HIVconsv immunogen (C). Data in panel B are represented as means ± standard errors of the means, *n* = 4 (mice); panel C shows means ± standard errors of the means, *n* = 3 (triplicate of pooled samples), with one-way ANOVA, *P* < 0.0001. (D) An independent experiment using the same CM regimen for immunization confirmed responses induced to the 8 peptides identified in panel C with the one-way ANOVA, *P* < 0.0001. (E) Mapping of minimal epitopes for peptide 42, corresponding to Gag amino acids 126 to 140, and peptide 112, corresponding to Pol amino acids 174 to 186/560-561 (containing a two-region junction), of the HXB2 sequence using progressively truncated peptides in an IFN-γ ELISPOT assay. Pooled splenocyte samples were employed as effectors.

We next assessed the magnitude, breadth, and functional spectrum of T-cell responses following the ChAdV63.HIVconsv-prime and MVA.HIVconsv-boost regimen (Fig. 2A), using 199 15-mer peptides overlapping by 11 amino acids (15/11) spanning the entire HIVconsv immunogen. These peptides were combined into pools P1 to P6 containing 32 to 35 individual 15/11 peptides and tested in an IFN-γ ELISPOT assay. High-magnitude responses to peptide pools P2, P4, and P6 were detected (Fig. 2B), thus further confirming the synergistic effect of ChAdV63.HIVconsv and MVA.HIVconsv vaccines. Mapping of the single peptides in each pool confirmed a high-frequency response to epitope H contained in peptides 197/198, which reached for the latter an average of 2,354 SFU/10^6^ splenocytes, and revealed two other dominant responses averaging 1,233 and 1,635 SFU/10^6^ cells for peptides 42 (Gag amino acids 126 to 140) and 112 (Pol amino acids 174 to 186/560-561, i.e., containing a two-region junction), respectively. Also, peptides 9, 15, 55, 151, and 164 induced weaker, but definite, positive vaccine-elicited responses (Fig. 2C), which may not have been detected using a less potent regimen (10, 22). These responses were confirmed in an independent experiment yielding average frequencies of 163, 210, 199, 90, and 180 SFU/10^6^ splenocytes, respectively (Fig. 2D). Peptide 9 (VGGHQAAMQMLKDTI) contains a known epitope; while the AMQMLK**E**TI is the index epitope (23, 24), the AMQMLK**D**TI variant present in the vaccine is much weaker (10, 24), which likely explains its low immunogenicity in these experiments. Peptides 42 (KAIGTVLVGPTPVNI), 151 (VHVASGYIEAEVIPA), and 164 (VQMAVFIHNFKRKGGI) do not contain any known epitope and are therefore considered novel. Optimal epitope mapping for peptides 42 and 112 using sequentially truncated peptides identified the respective 9-mer binding epitopes as LVGPTPVNI and YYDPSKDLI (Fig. 2E). Thus, the ChAdV63.HIVconsv-MVA.HIVconsv (CM) regimen elicited broadly specific T-cell responses.

### ChAdV63.HIVconsv-MVA.HIVconsv regimen induces oligofunctional T cells.

**FIG 3:**
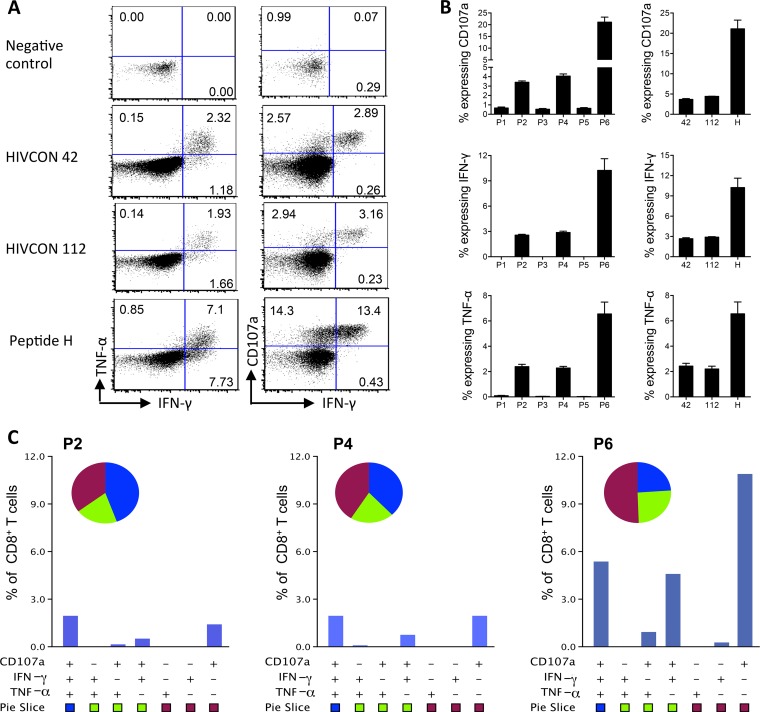
Functional characterization of T cells stimulated by the HIVconsv vaccines. BALB/c mice were vaccinated by the ChAdV63.HIVconsv-MVA.HIVconsv regimen. One week after MVA.HIVconsv, oligofunctional profiles of HIVconsv-elicited T cells were assessed by ICS assay. (A) Representative FACS plots for TNF-α or CD107a versus IFN-γ in response to peptides H, 42, and 112. (B) Frequencies of CD8^+^ T cells expressing TNF-α, CD107a, or IFN-γ upon stimulation using either peptide pools P1 to P6 (left) or individual peptides 42, 112, and H (right). Data are represented as means ± standard errors of the means (*n* = 3). (C) Oligofunctional profiles of CD8^+^ T cells specific for peptide pools P2, P4, and P6. Data are represented as means (*n* = 3). The intragroup variations were low as shown in panel B and were not plotted for this analysis.

**FIG 4:**
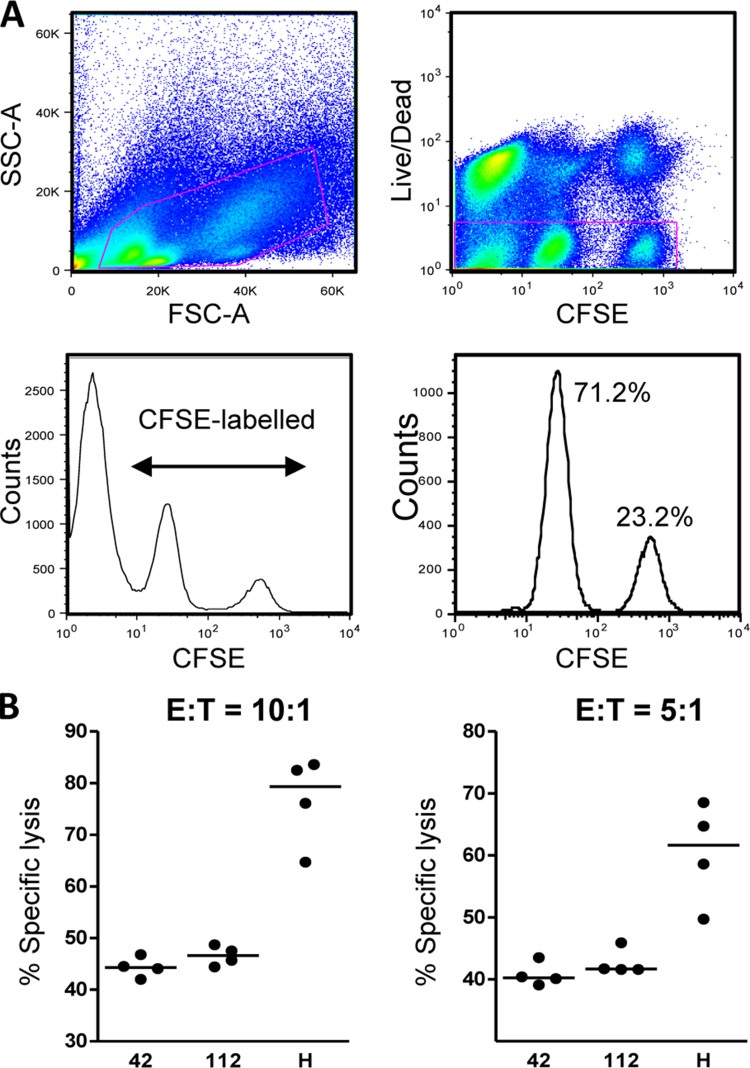
Cytotoxic potential of peptide 42-, 112-, and H-specific T cells induced by the HIVconsv vaccines. BALB/c mice were immunized with ChAdV63.HIVconsv followed by MVA.HIVconsv as shown in Fig. 2A. One week after MVA.HIVconsv, the splenocytes were tested for their potential to kill peptide-pulsed P815 cell targets. (A) Representative FACS plots showing the gating strategy. SSC, side scatter; FSC, forward scatter. (B) Percent specific lysis of peptide-pulsed P815 target cells at an effector-to-target ratio of 10:1 (left panel) or 5:1 (right panel). The horizontal lines represent the medians.

The functional diversity of HIVconsv-specific T cells elicited by the ChAdV63.HIVconsv-MVA.HIVconsv regimen was assessed using the ICS assay. Examples of dot plots for the 3 immunodominant epitopes are shown in Fig. 3A. As expected, the total frequencies of CD8^+^ T cells expressing CD107a, IFN-γ, or TNF-α functions were dominated by peptide pool P6 (Fig. 3B), which contains the immunodominant peptide H. This peptide pool showed a remarkably high frequency of specific degranulating CD107a^+^ CD8^+^ T cells, which exceeded 20% of the total CD8^+^ T cells. Strong responses to peptide pools P2 and P4 were also observed (Fig. 3B). Characterization of CD8^+^ T-cell polyfunctionality for peptide pools P2, P4, and P6 revealed that a large proportion of the responding cells expressed all three functions comprising CD107a, TNF-α, and IFN-γ (Fig. 3C). We further assessed the cytotoxic potential of HIVconsv-induced T cells in an *ex vivo* killing assay using peptide-pulsed cells as targets and demonstrated that freshly harvested, unstimulated splenocytes could efficiently kill peptide-pulsed P815 target cells, achieving medians of 44%, 47%, and 79% lysis of target cells sensitized with peptides 42, 112, and H, respectively, at an effector-to-target cell ratio of 10:1 (Fig. 4). These lysis data concur with the high frequency of degranulating CD107a^+^ CD8^+^ T cells observed in Fig. 3B and C and further define the functionality of HIVconsv-specific effector T cells.

## DISCUSSION

Nonreplicating vaccine vectors for delivery of pathogen-derived subunit immunogens are in the forefront of vaccine development for many infections. These vectors are most efficiently used in heterologous prime-boost regimens to avoid buildup of antivector antibodies, which dampen induction of immune responses against the transgene product (8). Although some general rules for combining heterologous vectors into a prime-boost regimen are emerging, optimization of vaccination regimens is mostly empirical. Incremental vaccination improvements are best assessed first in small-animal models such as the BALB/c mice.

This study indicated an ongoing expansion of ChAdV63.HIVconsv-elicited T-cell frequencies for at least 6 weeks postadministration. This was also reflected in the requirement for a long interval of 5 to 6 weeks after ChAdV63.HIVconsv priming to achieve high frequencies of HIVconsv-specific T cells by an MVA.HIVconsv boost and confirmed the impressive immunogenicity of a simple heterologous ChAdV63-prime and MVA-boost regimen observed in humans (16, 25, 26).

A comprehensive screening of peptides covering the entire HIVconsv immunogen identified several novel H-2^d^-restricted immunogenic regions recognized by the BALB/c mice. While the specificities of the stronger epitopes were described before (18, 27–29), responses to peptides 42, 151, and 164 would likely be missed using a vaccine delivery weaker than the CM regimen. This information increases the analysis “granularity” of T cells elicited by the conserved region as well as other HIV-1 vaccine candidates. More detailed and comprehensive study of cell-mediated responses aids further iterative improvements of the magnitude, breadth, functionality, and longevity of this important arm of immune defenses against microorganisms and may help shed light on more fundamental questions such as immunodominance and antigen processing.

Given the differences between the H-2^d^ and HLA major histocompatibility complex molecules, only some peptides immunogenic in the BALB/c mouse are also presented by the human HLAs. Thus, peptide H is unusual in that it has four anchor residues for H-2D^d^ (30, 31) and displays “promiscuous” binding to four different H-2D^d^, H-2D^p^, H-2^u^, and H-2^q^ murine determinants (32) as well as human HLA-A2 (33), although in our hands, in human volunteers (16, 34–43) or HLA-A2-transgenic HHD mice (unpublished data), such responses were never detected for either the HIVA (20) or the HIVconsv (9) immunogens. No responses were detected in 23 human recipients of the HIVconsv vaccine to peptide 42 or 112, while T cells were induced to peptides 151 and 164 (16). Although not directly comparable or translatable between mouse and humans, these results increase the confidence about the usefulness of optimizing new vaccine strategies first in a small and well-defined model.
